# Supplementary material for: Targeting EGFR-dependent tumors by disrupting an ARF6-mediated sorting system
Source: Nat Commun. 2022 Oct 12;13:6004. doi: 10.1038/s41467-022-33788-7 (PMC9556547; doi:10.1038/s41467-022-33788-7)
Supplement: Supplementary file 1 — Supplementary Information [file 41467_2022_33788_MOESM1_ESM.pdf]

Supplementary Information

Targeting EGFR-Dependent Tumors by Disrupting an ARF6-Mediated Sorting System

Huiling Guo[1,6], Juan Wang[2,6], Su Ren[1,6], Lang-Fan Zheng[2,6], Yi-Xuan Zhuang[1], Dong-Lin Li[1], Hui-Hui Sun[1], Li-Ying Liu[1], Changchuan Xie[1], Ya-Ying Wu[1], Hong-Rui Wang[1], Xianming Deng[1,3], Peng Li[2,4,5], Tong-Jin Zhao[2,5, *]

Supplementary Fig. 1

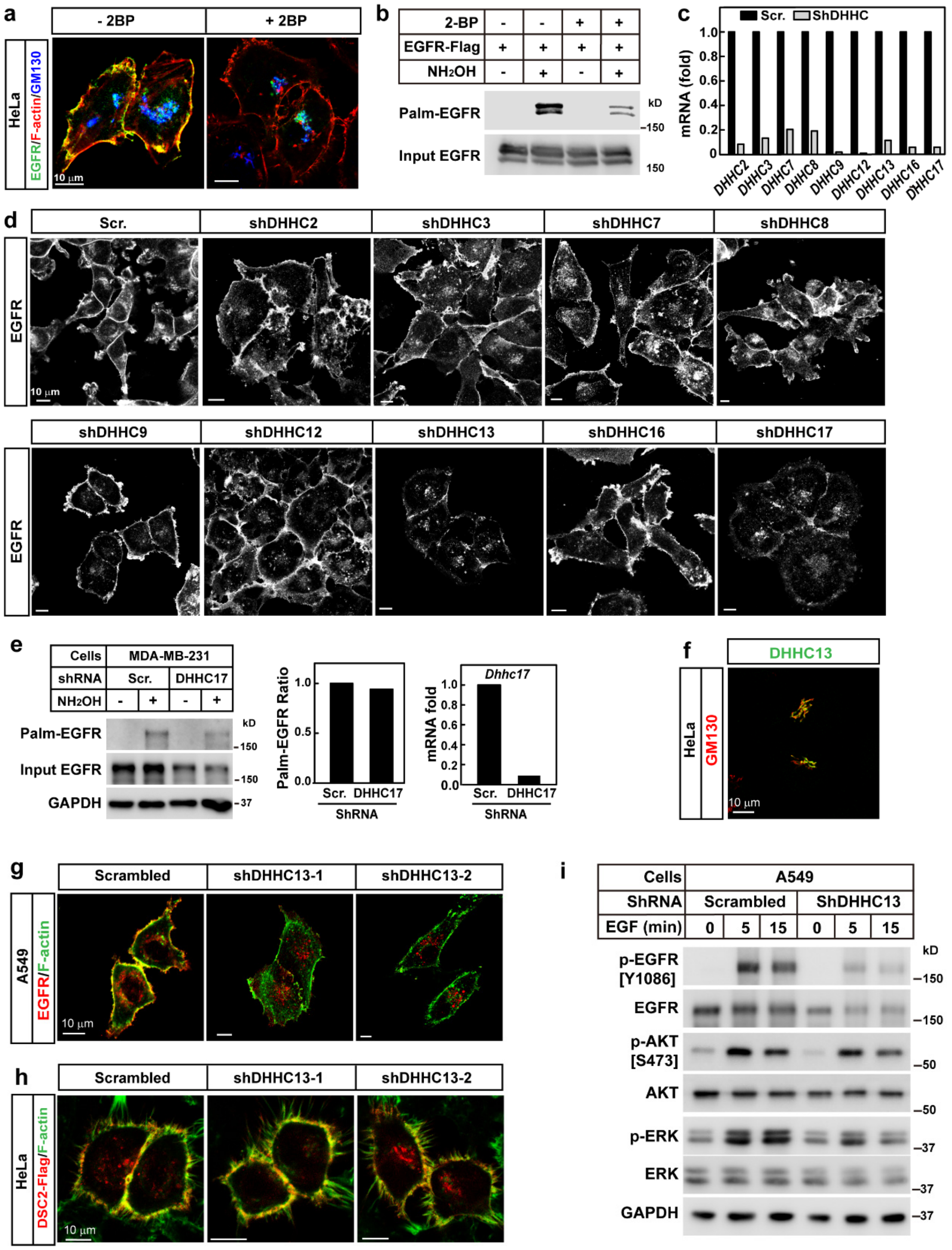

Supplementary Fig. 1. Palmitoylation is required for EGFR targeting to PM.

a,b, On day 0, HeLa cells were set up 2 x 10^4 cells per 35-mm dish and transfected with EGFR-Flag. On day 2, cells were treated with 100 µM 2-BP for 24 hr. On day 3, cells were harvested for immunostaining (a) and Acyl-RAC assay (b). Rhodamine-

labeled phalloidin was used in (a) to stain F-actin to indicate plasma membrane. **c,** Knockdown efficiency of the *DHHCs* in MDA-MB-231 cells. The expression level of each *DHHC* in scrambled shRNA-transduced cells is normalized to 1.0. *36B4* was used as a control. **d.** MDA-MB-231 cells expressing indicated shRNAs were set up and subjected into immunostaining with anti-EGFR antibody. Scale bar, 10 μm. **e,** On day 0, MDA-MB-231 cells were infected with lentivirus expressing scrambled shRNA or shDHHC17. On day 2, cells were selected with 2 μg/ml puromycin. On day 4, cells were subjected into Acyl-RAC assay. The intensities of indicated bands were quantified using the VisionWorks software on a ChemStudio imaging system and the ratios of palm-EGFR/input EGFR were shown in the lower left panel. Knockdown efficiency of *DHHC17* was shown in the lower right panel. **f,** HeLa cells were set up and transfected with DHHC13-Flag as in (a). On day 3, cells were harvested for immunostaining with anti-Flag and anti-GM130. **g,** Control and DHHC13 knockdown A549 cells were subjected to immunostaining as in Fig. 1d. Scale bar, 10 μm. **h,** DHHC13 knockdown HeLa cells were set up and transfected with DSC2-Flag as in (a). On day 3, cells were harvested for immunostaining with anti-Flag and FITC-labeled phalloidin. **i,** Control and DHHC13 knockdown A549 cells were pre-treated with serum-free medium overnight, followed by treatment with 100 ng/ml EGF for indicated time. Cells were then harvested and the lysates were subjected into western blot with indicated antibodies. Source data are provided as a Source Data file.

Supplementary Fig. 2

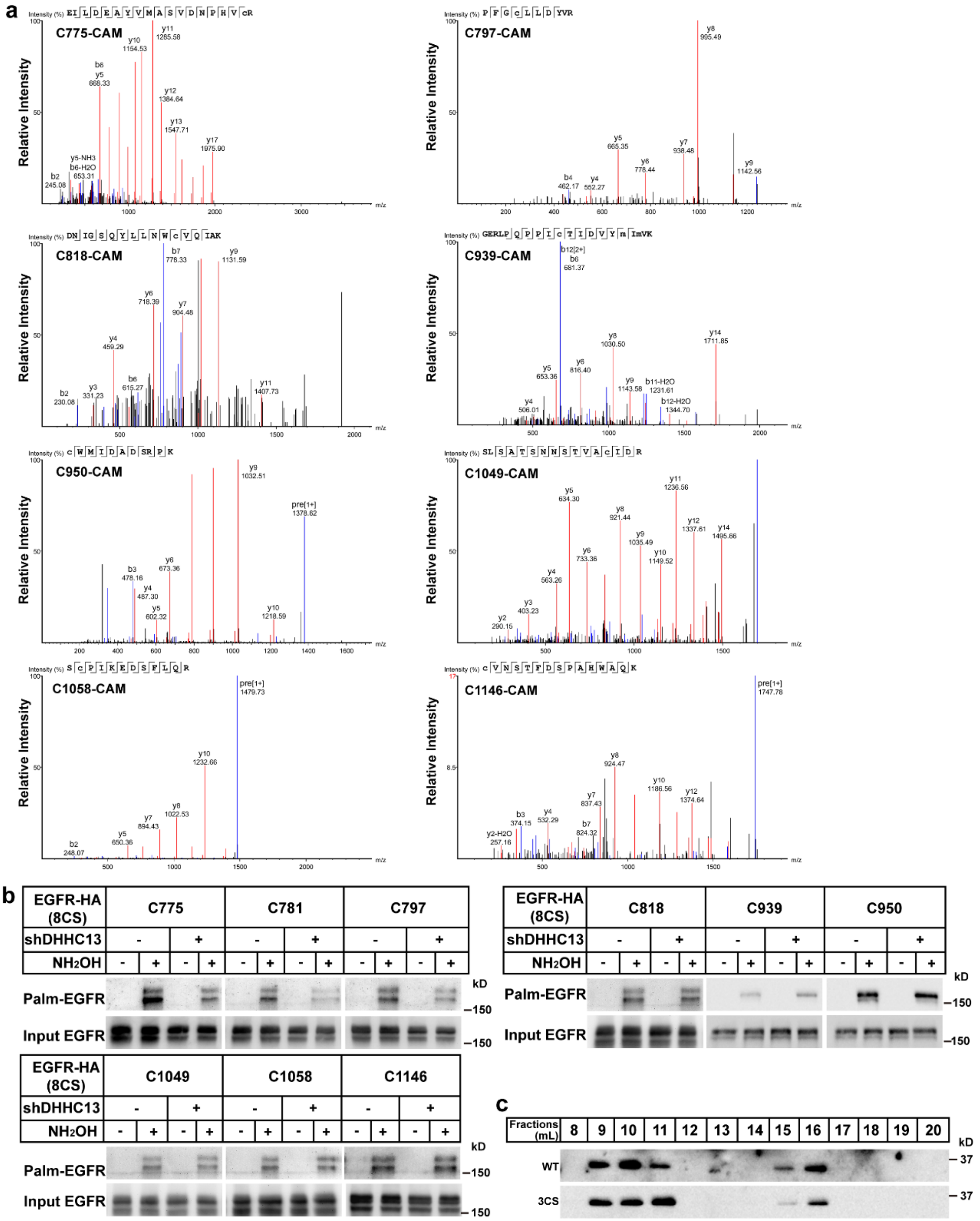

Supplementary Fig. 2. Cys775, Cys781 and Cys797 in EGFR are palmitoylated by DHHC13.

a, Identification of the palmitoylation sites of EGFR as described in the Methods. **b,** EGFR mutants with only one of the 9 cytosolic Cys (8CS) were generated and

introduced into control and DHHC13 knockdown HEK-293T cells for Acyl-RAC analysis. **c**, Flag-tagged WT and 3CS mutant of the EGFR cytosolic domain(696-960aa) were expressed in HEK-293T cells. Cells were lysed and the cytosolic fractions were analyzed on a gel filtration. Different fractions were collected and subjected to western blot. Source data are provided as a Source Data file.

Supplementary Fig. 3

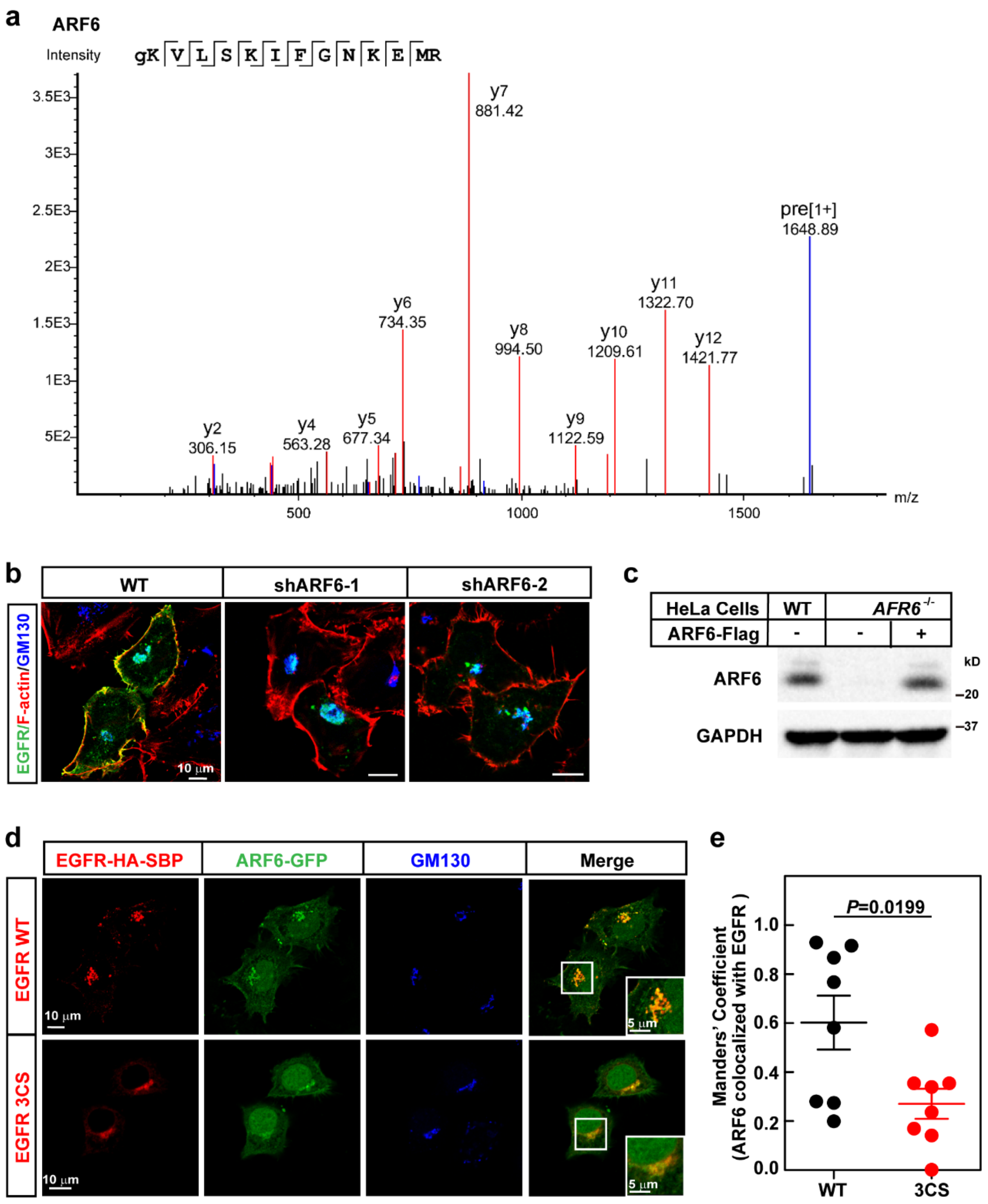

Supplementary Fig. 3. ARF6 is required for EGFR PM localization.

a. The bands from Fig. 2c were cut out and sent for mass spectrometry analysis. The spectrum of ARF6 peptides was shown. **b.** On day 0, HeLa cells infected with lentivirus expressing scrambled shRNA or shARF6 were set up as in supplementary Fig. 1a. On

day 2, cells were transfected with EGFR-Flag/pCDH-puro. On day 3, cells were harvested and subjected to immunostaining using anti-Flag and anti-GM130 antibodies. Rhodamine-labeled phalloidin was used to stain F-actin to indicate plasma membrane. Scale bar, 10 μm. **c.** Analysis of the Protein levels of ARF6 in WT and *ARF6*$^{-/-}$ HeLa cells. **d,e,** On day 0, *ARF6*$^{-/-}$ cells were transfected with Streptavidin-Golgin-84/pCDH-puro, WT or 3CS of EGFR-HA-SBP/pCDH-puro and ARF6-GFP/pCDH-puro (0.5 μg each). On day 1, cells were harvested and subjected into immunostaining (d). The colocalization of ARF6 and EGFR from 8 cells were analyzed by Image J and the values of Mander's Coefficient were showed in (e). The value represents mean ± SEM (n=8). Two-sided Student's t test was performed. Source data are provided as a Source Data file.

Supplementary Fig. 4

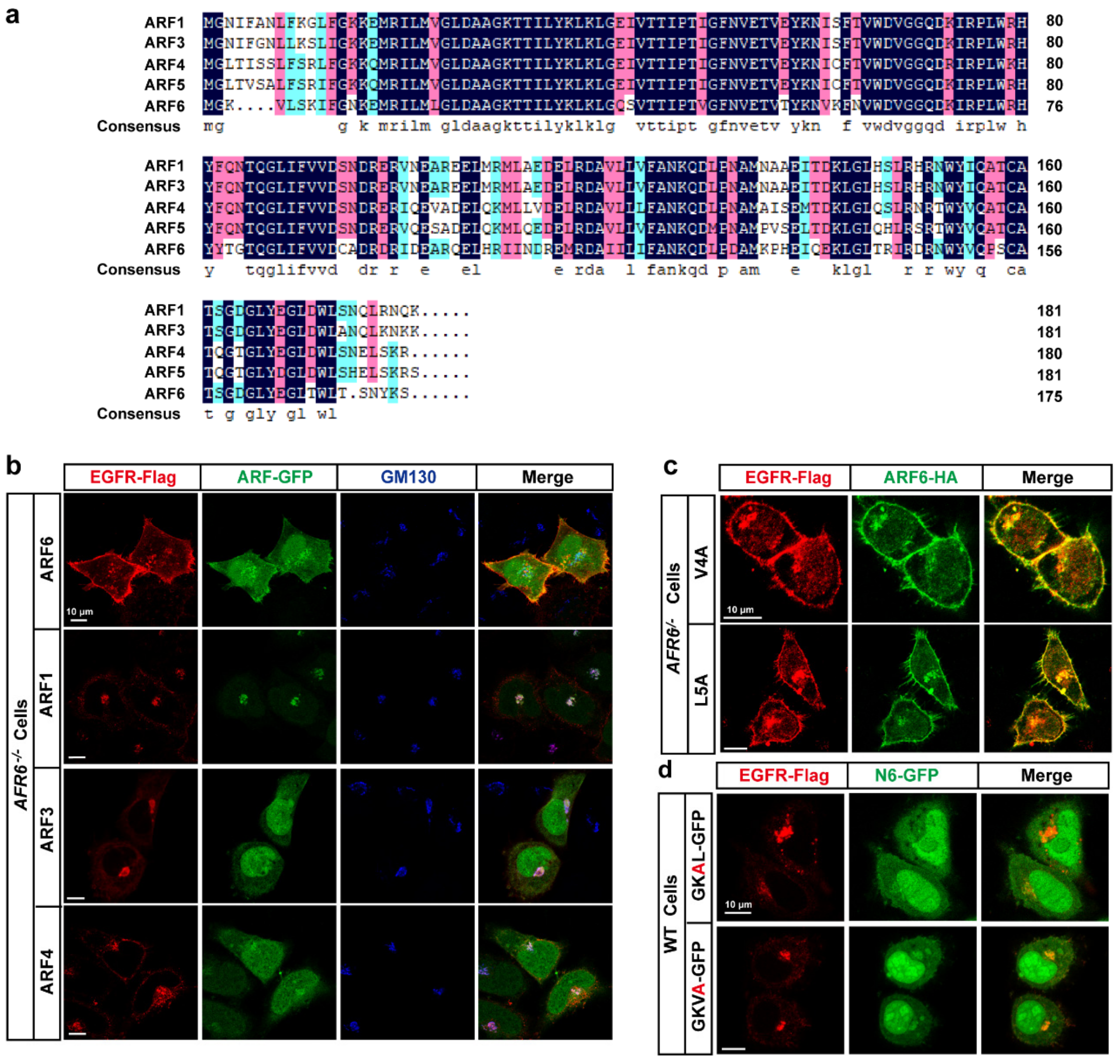

Supplementary Fig. 4. The N-terminus of ARF6 is required for targeting EGFR to PM.

A, Sequence alignment of 5 human ARF proteins. Alignment was performed using DNAMAN software. **b,** *ARF6*$^{-/-}$ HeLa cells transfected with EGFR-Flag and GFP-tagged ARF6, ARF1, ARF3, ARF4 (0.5 µg each) were serum-starved and subjected to immunostaining using anti-Flag and anti-GM130 antibodies as in Fig. 3a. Scale bar, 10 µm. **c,** The experiment was set up in the same way as in Fig. 3b except different mutants of ARF6 were used. Scale bar, 10 µm. **d.** The experiment was set up in the

same way as in Fig. 3c except that different GFP fusion proteins were used. Scale bar, 10 μm. Source data are provided as a Source Data file.

Supplementary Fig. 5

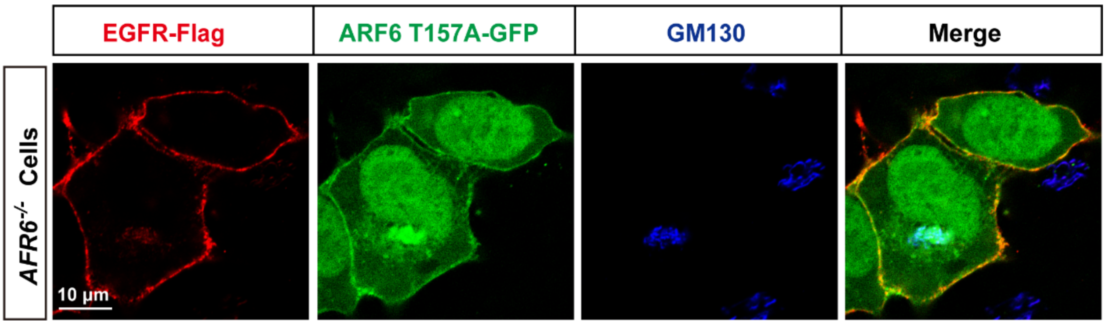

Supplementary Fig. 5. The GTP-bound form of ARF6 is required for targeting EGFR to PM.

On day 0, *ARF6*$^{-/-}$ HeLa cells were transfected with EGFR-Flag and T157A mutant of ARF6-GFP. On day 1, cells were switched to serum-free medium and subjected to immunostaining as in Fig. 3a. Scale bar, 10 μm.

Supplementary Fig. 6

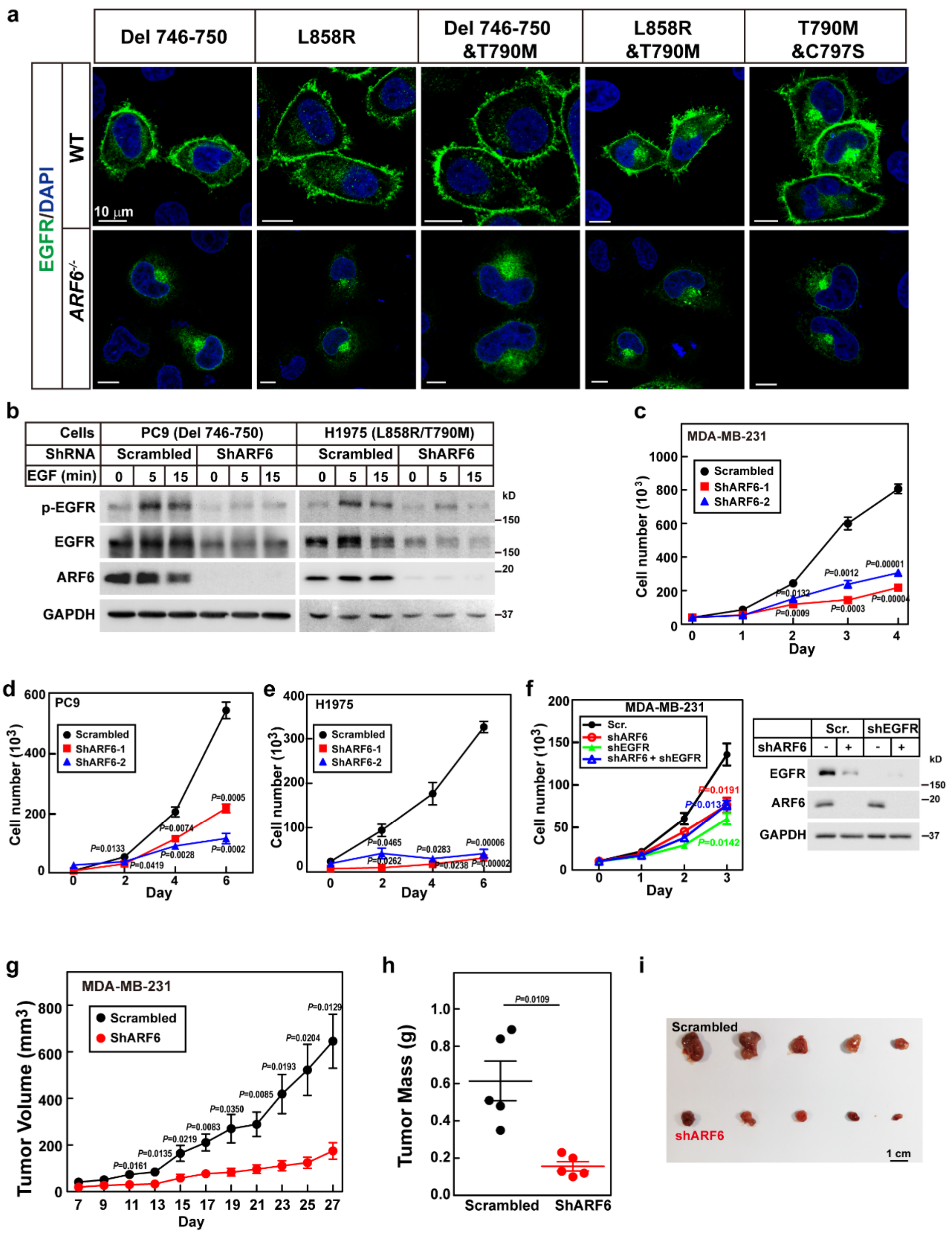

Supplementary Fig. 6 ARF6 is required for growth of tumors with aberrant EGFR activation.

a. WT and *ARF6*$^{-/-}$ HeLa cells were set up, transfected with the indicated mutants of EGFR, and harvested for immunostaining. Scale bar, 10 μm. **b.** Control and ARF6

knockdown PC9 or H1975 cells were set up, treated with EGF and harvested for immunoblotting as in Fig. 6b. **c-e,** On day 0, control (scrambled) and ARF6 knockdown MDA-MB-231 (c), PC9 (d) and H1975 (e) cells were set up at 4 x 10^4 cells per 35-mm dish. On the indicated days, 3 dishes cells were harvested and counted each day. Each value represent mean ± SEM of a triplicate. P-value denote the level of statistical significance (student's t test) between scrambled and ARF6 knockdown cells. **f**, MDA-MB-231 cells expressing indicated shRNAs were set up at 1 x 10^4 cells per 35-mm dish and harvested for cell count each day. Each value represent mean ± SEM of a triplicate. P-value denote the level of statistical significance (two-tailed paired t test) between scrambled and indicated shRNA expressing cells. Protein levels of EGFR and ARF6 in cells were confirmed by western blot in the right panel. **g-i,** On day 0, control and ARF6 knockdown MDA-MB-231 cells were subcutaneously implanted into the nude mice. For each nude mouse, control cells were implanted on the left side and ARF6 knockdown cells on the right side at 3 x 10^6 cells per spot. Starting from day 7, tumor size was measured every 2 days (g). On day 27, mice were dissected, tumor masses were weighted (h) and images were taken (i). Each value represents mean ± SEM of 5 samples. P-value denote the level of statistical significance (two-tailed paired t test) between scrambled and ARF6 knockdown tumors. Source data are provided as a Source Data file.

Supplementary Fig. 7

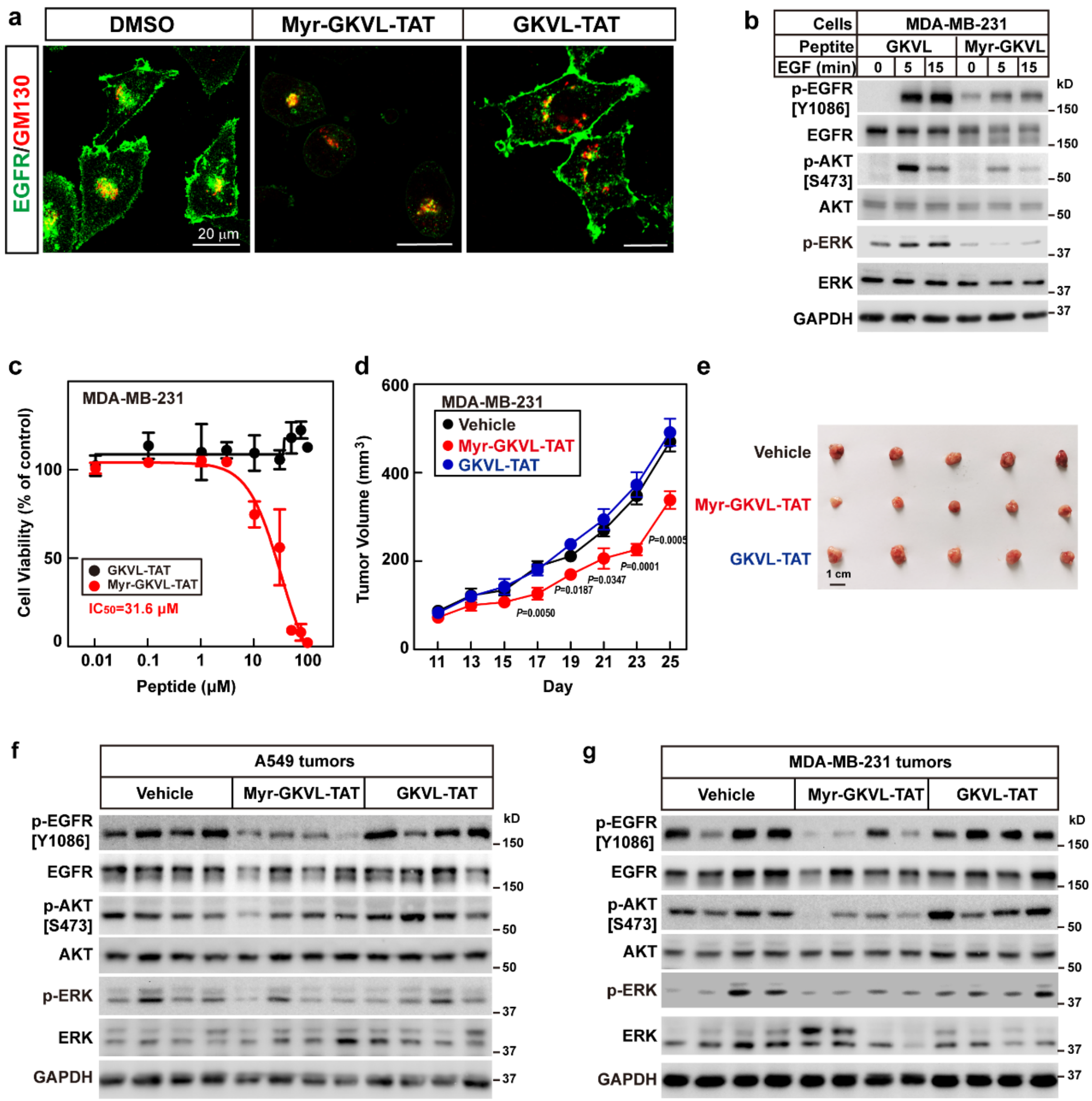

Supplementary Fig. 7 Myr-GKVL-TAT inhibits the growth of EGFR-overexpressing tumors.

a,b, On day 0, MDA-MB-231 cells were set up and treated with 10 μM Myr-GKVL-TAT or GKVL-TAT for 20 hr. Cells were then incubated with serum-free medium including the peptides for 4 hr. (**a**) Immunostaining assays were performed using the anti-EGFR antibodies. Scale bar, 20 μm. (**b**) Cells were treated with 100 ng/ml EGF, harvested at indicated time and subjected into western blot. **c.** On day 0, MDA-MB-231 cells were set up as in Fig. 7c. From day 1, cells were treated with various concentrations of Myr-

GKVL-TAT or GKVL-TAT for 2 days. On day 3, cell viability was determined by cell counting kit-8. Each value represent mean ± SEM of a triplicate. IC_{50} was analyzed using GraphPad Prism 5. **d, e.** On day 0, MDA-MB-231 cells were implanted into nude mice subcutaneously at 3 x 10^6 cells per mouse (n=8). Starting from day 11 when the tumors grew to around 100 mm^3, mice were treated with a daily subcutaneous injection of vehicle, Myr-GKVL-TAT (2 mg/kg) or GKVL-TAT (2 mg/kg). Tumor sizes were measured every two days (d). Each value represents mean ± SEM of 8 samples. P-value denote the level of statistical significance (two-tailed paired t test) between vehicle and Myr-GKVL-TAT treated tumors. On day 25, mice were euthanized and tumors were dissected (e). Representative images of mice and tumors were shown. **f,g,** Lysates from A549 (**f**) and MDA-MB-231 (**g**) tumors were subjected to western blot with indicated antibodies. Source data are provided as a Source Data file.

Supplementary Table 1. Primer information

Primers	Source of Primer sequences
**A. Targeted sequences of shRNAs and sgRNAs**	
hDHHC2-shRNA	GCCAAGGATCTTCCCATCTAT
hDHHC3-shRNA	GCTTTGAAGAAGATTGGACAA
hDHHC7-shRNA	GATAACTGTAATCCTGTTGAT
hDHHC8-shRNA	CACCTGCCATGTACAAGTTTA
hDHHC9-shRNA	GAGGAACTACCGCTACTTCTA
hDHHC12-shRNA	TTCATCTCCTCACACCGCATC
hDHHC13-shRNA-1	TACTCACTGATTATGGATAAA
hDHHC13-shRNA-2	CTCATGTTATCAGCTCACAAA
hDHHC16-shRNA	GAAAGACACAATGTGGAGAAA
hDHHC17-shRNA	GCAGGGAATACCACAGTCATT
hARF6-shRNA1	GCTCACATGGTTAACCTCTAA
hARF6-shRNA2	CAACAATCCTGTACAAGTTGA
hARF6-sgRNA1	GCGGCCGAGAGGCTTCGTTT
hARF6-sgRNA2	TCTACAGTTTGGCGGGGACG
hEGFR-shRNA	GCCACAAAGCAGTGAATTTAT
hEXOC2-shRNA	CTTGAGACACCATCAACTTTA
hEXOC5-shRNA	GCCAGCTGATTCAGGAGTTTA
hEXOC6-shRNA	GCTCAACAGAAATAGACGATA
hEFA6B-shRNA	TTCCCTGTGCCCATCTATAAA
hEFA6D-shRNA	CTGTGCAATAATGCTTCTTAA
hGEP100-shRNA	CCTTCTCTAGGCAAGTGAAAT
B. **Primers to generate different constructs**	
hEGFR-F	CTAGTCTAGAATGCGACCCTCCGGGACGGC
hEGFR-R	CCGCTCGAGTGCTCCAATAAATTCACTGC
EGFR-C797S-F	CCCTTCGGCTCCCTCCTGGACTATGTCCGGGAA
EGFR-C797S-R	GTCCAGGAGGGAGCCGAAGGGCATGAGCTGCGT
EGFR C775S C781S-F	CCCCACGTGTCCCGCCTGCTGGGCATCTCCCTC
EGFR C775S C781S-R	GGAGGTGAGGGAGATGCCCAGCAGGCGGGACAC
EGFR C818S-F	GCTCAACTGGTCTGTGCAGATCGCAAAGGGCAT
EGFR C818S-R	GATCTGCACAGACCAGTTGAGCAGGTACTGGGA
EGFR C939S C950S-F	CCACCCATATCTACCATCGATGTCTACATGATCATGGTCAAGTCC
EGFR C939S C950S-R	CTATCATCCAGGACTTGACCATGATCATGTAGACATCGATGGTAGA
EGFR C1049S C1058S-F	ACCGTGGCTTCCATTGATAGAAATGGGCTGCAAAGCTCTCCC
EGFR C1049S C1058S-R	CTTGATGGGAGAGCTTTGCAGCCCATTTCTATCAATGGAAGC

EGFR C1146S-F	CAGCCCACCTCTGTCAACAGCACATTCGACAGC
EGFR C1146S-R	GCTGTTGACAGAGGTGGGCTGGACAGTGTTGAG
EGFR-C775-F	GACAACCCCCACGTGTGCCGCCTGCTGGGCATC
EGFR-C775-R	CAGCAGGCGGCACACGTGGGGGTTGTCCACGCT
EGFR-C781-F	CTGCTGGGCATCTGCCTCACCTCCACCGTGCAG
EGFR-C781-R	GGTGGAGGTGAGGCAGATGCCCAGCAGGCGGCA
EGFR-C797-F	ATGCCCTTCGGCTGCCTCCTGGACTATGTCCGG
EGFR-C797-R	ATAGTCCAGGAGGCAGCCGAAGGGCATGAGCTG
EGFR-C818-F	CTGCTCAACTGGTGTGTGCAGATCGCAAAGGGC
EGFR-C818-R	TGCGATCTGCACACACCAGTTGAGCAGGTACTG
EGFR-C939-F	CAGCCACCCATATGTACCATCGATGTCTACATG
EGFR-C939-R	GACATCGATGGTACATATGGGTGGCTGAGGGAG
EGFR-C950-F	ATCATGGTCAAGTGCTGGATGATAGACGCAGAT
EGFR-C950-R	GTCTATCATCCAGCACTTGACCATGATCATGTA
EGFR-C1049-F	TCCACCGTGGCTTGCATTGATAGAAATGGGCTG
EGFR-C1049-R	ATTTCTATCAATGCAAGCCACGGTGGAATTGTT
EGFR-C1058-F	GGGCTGCAAAGCTGTCCCATCAAGGAAGACAGC
EGFR-C1058-R	TTCCTTGATGGGACAGCTTTGCAGCCCATTTCT
EGFR-C1146-F	GTCCAGCCCACCTGTGTCAACAGCACATTCGAC
EGFR-C1146-R	TGTGCTGTTGACACAGGTGGGCTGGACAGTGTT
hARF1-F	AGAGCTAGCGAATTCATGGGGAACATCTTCGCCAAC
hARF1-R	GTCGGATCCCTCGAGCTTCTGGTTCCGGAGCTGATT
hARF3-F	AGAGCTAGCGAATTCATGGGCAATATCTTTGGAAAC
hARF3-R	GTCGGATCCCTCGAGCTTCTTGTTTTTGAGCTGATT
hARF4-F	AGAGCTAGCGAATTCATGGGCCTCACTATCTCCTCC
hARF4-R	GTCGGATCCCTCGAGACGTTTTGAAAGCTCATTTGA
hARF5-F	CATAGAATTCATGGGCCTCACCGTGTCCG
hARF5-R	CTTACTCGAGGCGCTTTGACAGCTCGTGG
hARF6-F	CCGGAATTCATGGGGAAGGTGCTATCC
hARF6-R	CCGCTCGAGAGATTTGTAGTTAGAGGTTA
N5C6-F	CATAGAATTCATGGGGCTCACCGTGTCCGCGGTGCTATCCAAAATCTTCG
N6C5-F	CATAGAATTCATGGGCAAGCTCTTTTCGCGGATCTTCGG
hARF6-G2A-F	CCGGAATTCATGGCGAAGGTGCTATCC
hARF6-K3A-F	CCGGAATTCATGGGGGCGGTGCTATCCAA
hARF6-V4A-F	CCGGAATTCATGGGGAAGGCGCTATCCAA
hARF6-L5A-F	CCGGAATTCATGGGGAAGGTGGCGTCCAA
hARF6-T27N-F	GCCGGCAAGAACACAATCCTGTACAAGTTGA
hARF6-T27N-R	CAGGATTGTG TTCTTGCCGG CCGCGTCCAGGC
hARF6-Q67L-F	GTGGGCGGCCTCGACAAGATCCGGCCGCTCTG
hARF6-Q67L-R	GATCTTGTCG AGGCCGCCCA CATCCCATACGT

hARF6-T157A-R	GTCGGATCCCTCGAGAGATTTGTAGTTAGAGGTTAACC ATGTGAGCCCCTCATAGAGTCCGTCCCCTGAGGCGGC
GKVL-GFP-F	CTAGCTAGCATGGGGAAGGTGCTAGTGAGCAAGGGCG AGGA
AKVL-GFP-F	CTAGCTAGCATGGCGAAGGTGCTAGTGAGCAAGGGCG AGGA
GAVL-GFP-F	CTAGCTAGCATGGGGGCGGTGCTAGTGAGCAAGGGCG AGGA
GKAL-GFP-F	CTAGCTAGCATGGGGAAGGCGCTAGTGAGCAAGGGCG AGGA
GKVA-GFP-F	CTAGCTAGCATGGGGAAGGTGGCAGTGAGCAAGGGCG AGGA
GFP-R	CCGGAATTCTGATCTAGAGTCGCGGCCGC
hEXOC1-F	CCGCTCGAGACAGCAATCAAGCATGCATT
hEXOC1-R	CGATACCGGTTTAGTGGGACTGTGCAATGC
hEXOC2-F	CCGCTCGAGTCTCGATCACGACAACCCCC
hEXOC2-R	CGATACCGGTTTATGTTTTCATCATGGTTG
hEXOC3-F	CCGCTCGAGAAGGAGACAGACCGGGAGGC
hEXOC3-R	CGATACCGGTCTACTTGAGCAGCTTGGCCA
hEXOC4-F	CCGCTCGAGGCGGCAGAAGCAGCTGGTGG
hEXOC4-R	CGATACCGGTCTAAACGGTAGTTATCTTCT
hEXOC5-F	CGCGGATCCGCTACCACGGCCGAGCTCTT
hEXOC5-R	CCGCTCGAGTCAGCTGAAGTGTCGAGCAA
hEXOC6-F	CGCGGATCCGCGGAGAACAGCGAGAGTCT
hEXOC6-R	CCGCTCGAGCTACATGTGCTGGGACATAC
hEXOC7-F	CACCGGATCCATTCCCCCACAGGAGGCATC
hEXOC7-R	CACCCTCGAGTCAGGCAGAGGTGTCGAAAAG
hEXOC8-F	CCGGAATTCGCGATGGCGATGTCGGACAG
hEXOC8-R	CGATACCGGTTTAGACCACTGATGTTGTAC
hEXOC2-shRNA	CTTGAGACACCATCAACTTTA
hEXOC5-shRNA	GCCAGCTGATTCAGGAGTTTA
hEXOC6-shRNA	GCTCAACAGAAATAGACGATA
hEFA6B-shRNA	TTCCCTGTGCCCATCTATAAA
hEFA6D-shRNA	CTGTGCAATAATGCTTCTTAA
hGEP100-shRNA	CCTTCTCTAGGCAAGTGAAAT
hCytohesin1-shRNA	CCCTTTAGAGAATCTGAGTAT
hCytohesin2-shRNA	CAGTTCTTGGTGGAGAATGAA
hCytohesin3-shRNA	CCCTGGTTCATATTTGAGTTT
hEFA6B-F	AGAGCTAGCGAATTCATGATGGGTGACTACAGA
hEFA6B-R	TTTGTAGTCGGATCCCAGCTGATTGCGGTTCCG
**C. Quantitative real-time PCR Primers**	
h*DHHC*2	TCCCGGTGGTGTTCATCAC; CAACTTGTTCGCCAGTGTTTTC

h*DHHC*3	AGCATCATCAACGGAATTGTGT;<br>CCGAATGCACCGCTTACAAAC
h*DHHC*7	CCCAAAGGAAACGCTACGAAA;<br>CGCGCTCGGGTTTAATACAG
h*DHHC*8	ATCATGTGGCCCTGCAGCCCCTGCG;<br>TTCACACCGAGATCTCGTAGGTGGTC
h*DHHC*9	CCTGGGTGGGGAATTGTGTT;<br>ACGACGGACCAGAGTGTAAAG
h*DHHC*12	GTGCTGACCTGGGGAATCAC;<br>CTGCACATTCACGTAGCCA
h*DHHC*13	GGCCTGACCTCCCATGAGA;<br>CTGGCTGGGTGAAAGACCAT
h*DHHC*16	TGATGCTGCCTTTGAGCCTGTC;<br>GCACTGAGTAGGTTCGGAGGAT
h*DHHC*17	GATGTACGGCAACCGGACAAA;<br>TGATCCACAATAGCACCTTTCG
h*Efa6b*	TCAACCTGTACTTGGGAGACA;<br>GCCCCAGGGAGGAACATTTT
h*Efa6d*	AACACGGCTAGAAGCTCATTC;<br>TCCAGCGTCATTCCTGTAAAATC
h*Gep100*	CCGGATCACTACGAGCACAC;<br>CCCCATACTTTCGTTCTAGCATC
h*Cytohesin1*	TGCTGGCTGACATTCAGAGG;<br>TTGTTGAGCCCTTCGCCTTT
h*Cytohesin2*	AGTTCACCGACCTCAATCTGG;<br>CACAGGCAGTATCGCTGGG
h*Cytohesin3*	GGAGAGCAAAACGACTCAGAG;<br>CCCCAATGACGGTCTTATTTAGG
h*Exoc2*	TAACCCGCTTGGCATTGAGAT;<br>CCTCACTCTTCTTGTTAGCCTGT
h*Exoc5*	TCCGATGCAGAGCAATATCTCA;<br>GCGCTGTAGGATCATAGCACTT
h*Exoc6*	ACCGAAGGTTTCAAGATGCTG;<br>CACAGGAAGGCATAACTGCAA
