# Supplementary material for: Targeting EGFR-dependent tumors by disrupting an ARF6-mediated sorting system
Source: Nat Commun. 2022 Oct 12;13:6004. doi: 10.1038/s41467-022-33788-7 (PMC9556547; doi:10.1038/s41467-022-33788-7)
Supplement: Supplementary file 3 — Description of Additional Supplementary Files [file 41467_2022_33788_MOESM3_ESM.pdf]

Description of Additional Supplementary Files

File Name: Supplementary Data 1
Description: Contains the raw mass spectrometry data for Fig. 2c, Fig. 4a and Supplementary Fig. 2a.
